# Amyotrophic Lateral Sclerosis Risk, Family Income, and Fish Consumption Estimates of Mercury and Omega-3 PUFAs in the United States

**DOI:** 10.3390/ijerph18094528

**Published:** 2021-04-24

**Authors:** Hannah I. Hoffman, Walter G. Bradley, Celia Y. Chen, Erik P. Pioro, Elijah W. Stommel, Angeline S. Andrew

**Affiliations:** 1 Department of Biological Sciences, Dartmouth College, Hanover, NH 03755, USA; hoffmanh@mit.edu (H.I.H.); celia.y.chen@dartmouth.edu (C.Y.C.); 2 Department of Neurology, Miller School of Medicine, University of Miami, Miami, FL 33146, USA; w.bradley@med.miami.edu; 3 ALS and Neuromuscular Disease Center, Cleveland Clinic, Cleveland, OH 44195, USA; pioroe@ccf.org; 4 Department of Neurology, Geisel School of Medicine, Lebanon, NH 03756, USA; Elijah.W.Stommel@hitchcock.org

**Keywords:** amyotrophic lateral sclerosis, mercury, omega-3 fatty acids, fish, neuromuscular disease

## Abstract

Most amyotrophic lateral sclerosis (ALS) cases are considered sporadic, without a known genetic basis, and lifestyle factors are suspected to play an etiologic role. We previously observed increased risk of ALS associated with high nail mercury levels as an exposure biomarker and thus hypothesized that mercury exposure via fish consumption patterns increases ALS risk. Lifestyle surveys were obtained from ALS patients (*n* = 165) and *n* = 330 age- and sex-matched controls without ALS enrolled in New Hampshire, Vermont, or Ohio, USA. We estimated their annual intake of mercury and omega-3 polyunsaturated fatty acid (PUFA) via self-reported seafood consumption habits, including species and frequency. In our multivariable model, family income showed a significant positive association with ALS risk (*p* = 0.0003, adjusted for age, sex, family history, education, and race). Neither the estimated annual mercury nor omega-3 PUFA intakes via seafood were associated with ALS risk. ALS incidence is associated with socioeconomic status; however, consistent with a prior international study, this relationship is not linked to mercury intake estimated via fish or seafood consumption patterns.

## 1. Introduction

Amyotrophic lateral sclerosis (ALS) is a progressive neurological disease that damages motor neurons and can induce severe disability. While approximately 10% of patients inherit forms of ALS that are familial, lifestyle and environmental factors likely play a role in many cases [1]. We previously used biomarkers in toenail and fingernail tissue to link mercury exposure to increased risk of ALS in two independent U.S. cohorts [2,3]. The source of this mercury exposure remains unclear.

While mercury is thought to be a risk factor for the disease, omega-3 polyunsaturated fatty acids (PUFA) could be protective factors against ALS, as food frequency questionnaire estimates showed that omega-3 PUFA intake was associated with reduced risk in a large prospective cohort analysis [4]. The leading source of both mercury and omega-3 PUFA exposure in the United States is seafood consumption, as seafood species concentrate chemicals in their viscera and muscle tissue through ingestion of algae and seafood species in lower trophic levels [5,6,7,8]. Humans retain up to 95% of these chemicals per bite of seafood [9]. Therefore, we hypothesized that the source of the mercury that is associated with increased risk of ALS could be seafood. Since omega-3 PUFA might be protective against the effect of mercury, we assessed the intake of omega-3 PUFA via seafood. Also, in a nationally distributed control cohort, we observed previously that the toenail mercury levels were positively associated with income level, with a mean per-person household income of ~$56,000 for those in the top decile for toenail mercury, vs. ~$38,000 for those with lower mercury levels [3]. We then postulated that fish consumption patterns, particularly, frequent consumption of costly high-trophic level species that have high levels of mercury may be linked to ALS risk. Hence, we investigated the links between ALS and socioeconomic status and mercury intake via fish consumption.

Using demographic and seafood consumption questionnaire data from participants in our case–control studies in Northern New England and Ohio, we assessed the relationship between socioeconomic status, estimated mercury, and omega-3 PUFA intake and ALS risk.

## 2. Materials and Methods

### 2.1. ALS Patients

Were enrolled through medical centers and clinics in New Hampshire, Vermont, and Ohio, as described previously [10]. Participants were required to be at least 21 years of age and residents of Northern New England or Ohio (comprising the states of New Hampshire, Vermont, Maine, or Ohio) at the time of enrollment (2016–2020). The eligible ALS patients were newly diagnosed cases with either definite or probable ALS according to the Awaji-modified El Escorial criteria [11]. Eligible diagnoses included progressive bulbar palsy, but not primary muscular atrophy (PMA) or primary lateral sclerosis (PLS).

### 2.2. The Population-Control Participants

Selected from among the same catchment counties as the cases, were identified as residents of New Hampshire, Vermont, or Ohio using the U.S. Postal Service Delivery Sequence file licensed to Marketing Systems Group (Horsham, PA, USA). The sampling algorithm was designed based on the expected demographic distribution of the ALS cases, with over-sampling of males and 50–75-year-old subjects. Questionnaires were mailed out to the sampled individuals, followed by a postcard reminder. Questionnaire response rates were 56% for ALS patients and 10% for the population controls. Participants who returned a completed questionnaire received a $20 reimbursement. Participants consented to join the study. All study procedures were approved by the local human subjects protection committees at each contributing institution.

We used the R-package “MatchIt” to perform propensity score matching with a 2:1 ratio to select a subset of *n* = 330 population control questionnaires as a comparison group with a similar age and gender distribution to that of the ALS cases group (*n* = 165 Awaji definite or probable) [12]. This procedure selects two controls with the nearest age and same gender as each case.

### 2.3. Estimating Mercury Consumption

We used these surveys to estimate the annual mercury consumption among seafood consumers by cross-referencing self-reported consumption of seafood by type with the corresponding species-specific mean mercury concentrations and multiplying this value by the consumption frequency. Fish or seafood type was recorded in a free response chart, broken down into those types consumed weekly, monthly, or annually. Species-specific mean mercury concentrations were primarily based on previously published U.S. market means [13,14,15,16,17,18,19,20]. Several fish species required consulting additional sources to obtain a mercury concentration value [21,22,23,24].

### 2.4. Estimating Omega-3 PUFA Consumption

We also used published U.S. market surveys to estimate the annual omega-3 PUFA consumption among seafood consumers with a similar tactic: cross-referencing self-reported consumption of seafood by type with the corresponding species-specific mean omega-3 PUFA concentrations and multiplying this value by the consumption frequency (recorded as weekly, monthly, or annually) [23,24,25,26,27,28]. Species-specific mean omega-3 PUFA concentrations were primarily based on U.S. market means (Supplemental Table S1). Several species required consulting additional omega-3 PUFA concentration references (Supplementary Table S2).

### 2.5. Statistical Analysis

We started by testing for univariate associations between the response variable, ALS case–control status of participants, and categorical predictor variables by utilizing the chi-square test of independence and Fisher’s exact test. Multivariable modeling used case–control status as the outcome in an unconditional logistic regression analysis with adjustment for the potential confounders age, gender, family history, and smoking status. The index year was defined as the year of diagnosis for ALS patients or an equivalent year for controls. Bayesian Kernal Mixture Modeling (BKMR) was performed to assess the possible interactions between income, mercury, and omega-3 PUFA estimates [29]. These analyses were all performed using R: A Language and Environment for Statistical Computing, version 4.02 (R Foundation for Statistical Computing, Vienna, Austria).

### 2.6. Data Availability

The database tabulating mean mercury and omega-3 polyunsaturated fatty acid (PUFA) concentrations in seafoods is provided in Supplemental Table S1.

## 3. Results

ALS patients (*n* = 165) and their 2:1 matched controls were similar for age, gender, race, smoking, as well as educational attainment (Table 1). Approximately half of the ALS patients were in the age range of 50–65 years. ALS was present in the family history of 10.9% of the patients and almost none of the controls (*p* < 0.001). All ALS patients met the Awaji criteria for definite or probable ALS. Approximately 50% of the population reported consuming fish regularly (at least 15 times a year), and this overall consumption was not associated with ALS risk (*p* = 0.68).

Table 2 shows the multivariable model we constructed, containing age, sex, smoking, family history, family income, education, and race. Family history of ALS increased risk of ALS nearly 4-fold. A family income $150,000 or over was associated with a 2-fold higher risk of ALS, compared to those in the $60–80,000 income bracket.

**Figure 1:**
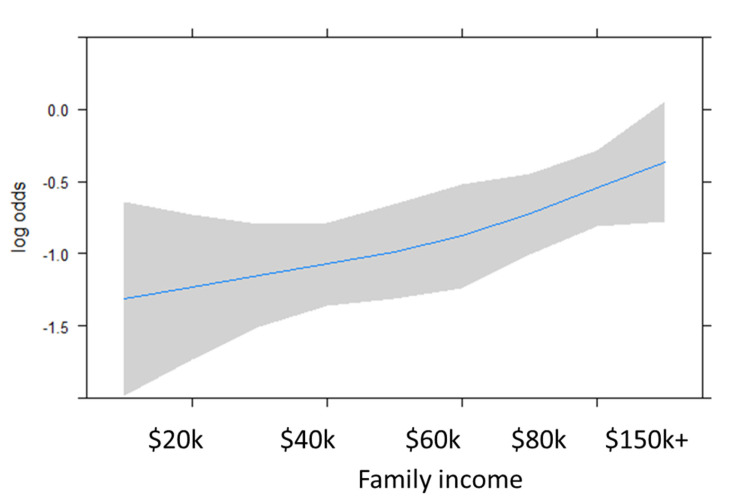
Spline model depicting the risk of ALS by family income (*p* = 0.00030). Blue line represents the log odds and the grey shading depicts the 95% CI.

In contrast, family income was positively associated with risk of ALS, increasing 23% for every $20,000 in income through $80,000 (OR 1.23 95% CI 1.10–1.38, adjusted for age, sex, family history, education, and race) (Figure 1).

**Figure 2:**
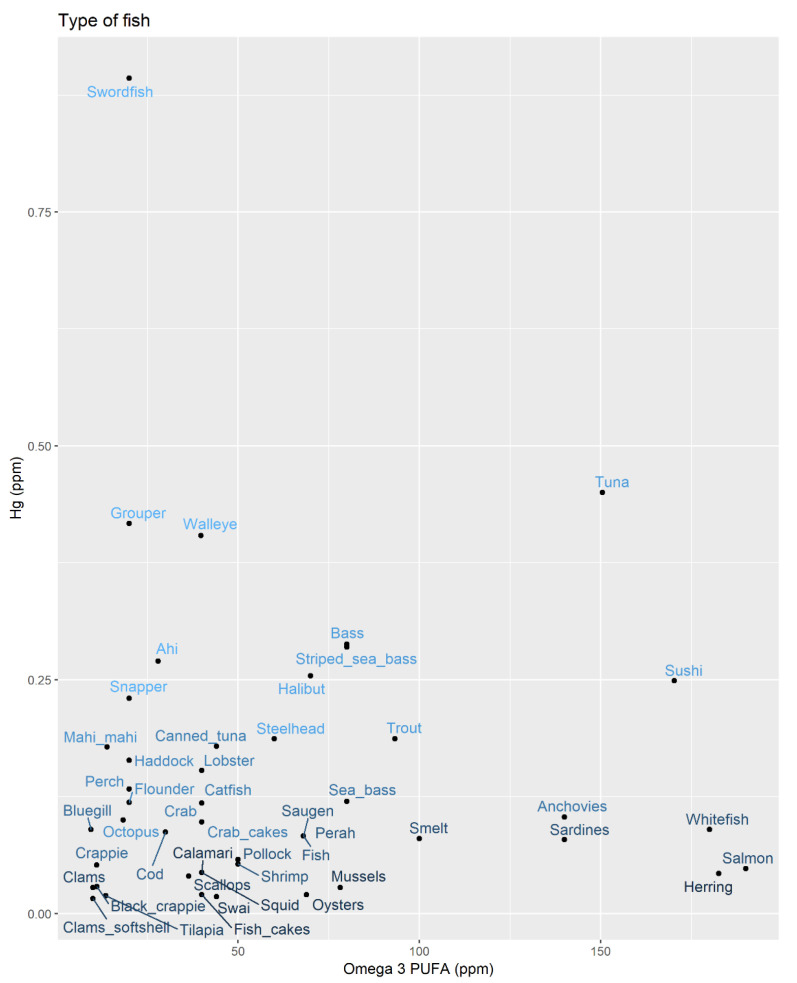
Mean mercury and omega-3 polyunsaturated fatty acid (PUFA) concentrations in seafood consumed by study participants. (brighter blue indicates higher mercury levels).

We used a fish-consumption questionnaire to estimate the annual intake of mercury and omega-3 PUFA by multiplying the content of a typical fish of each species by the frequency. Figure 2 shows the relationship between mercury and omega-3 PUFA content by fish type. The two are generally co-occurring, with only a few outlying species (e.g., swordfish) having very high mercury content, but low omega-3 PUFA. Whitefish, salmon and herring have the lowest mercury content, but high omega-3 PUFA.

As shown in Table 3, among the ~50% of the population who eat fish regularly, the log of the mean estimated annual consumption of neither mercury (*p* = 0.82) nor of omega-3 PUFA (*p* = 0.74) were associated with ALS risk.

**Figure 3:**
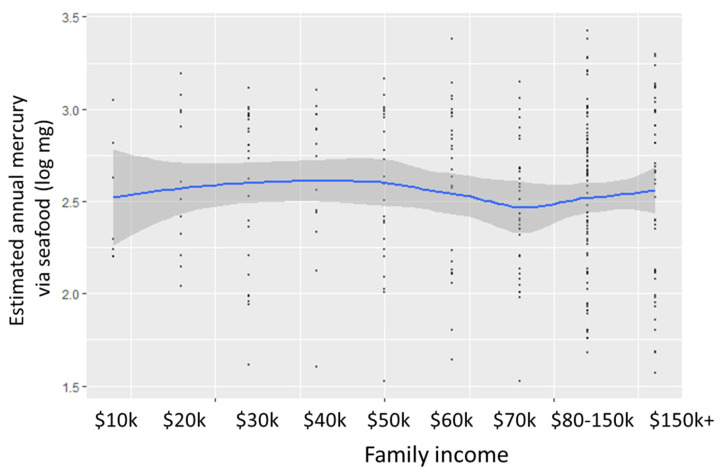
Annual mercury intake by family income.

Contrary to our hypothesis, our estimate of the annual mercury intake via seafood was not related to income (Figure 3).

**Figure 4:**
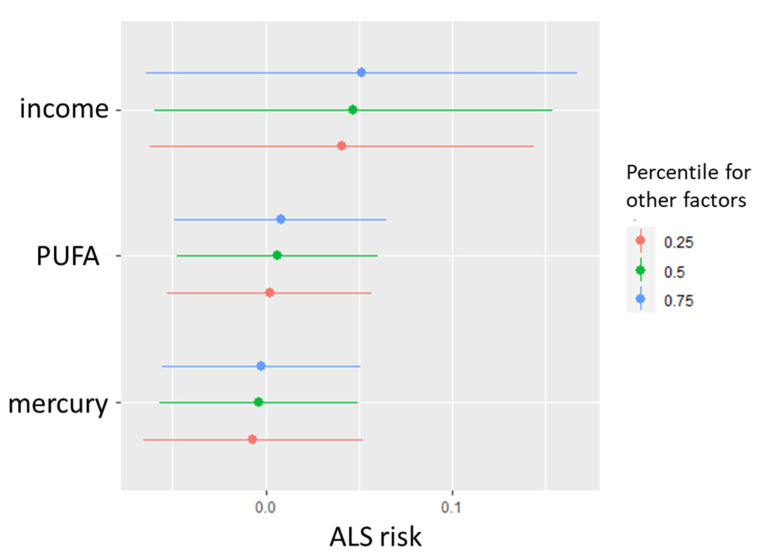
Relationship between income and both estimated mercury and omega-3 PUFA intakes via fish and seafood.

Figure 4 shows a Bayesian Kernal Mixture Model of income, intake of mercury, and omega-3 PUFA in relation to ALS risk. Mercury and omega-3 PUFA intakes did not show a relationship with ALS risk, hovering around 0.0. The increased risk associated with income did not vary by 25th, 50th, and 75th quartiles of mercury and omega-3 PUFA intake.

## 4. Discussion

Previous studies support the association between ALS diagnosis and mercury levels in biomarkers. In Japan, participants with ALS had greater hair mercury concentrations than control participants [30]. In our prior work in Northern New England, U.S. participants with ALS had higher toenail mercury levels than controls, adjusting for age and sex [2]. We replicated this relationship using nails from nationally distributed U.S. cohorts [3]. However, these studies did not identify the source of this mercury exposure.

Within the nationally distributed Sister Study cohort of controls without neurodegenerative illness, higher income was positively associated with toenail mercury biomarker levels [3]. Similarly, the National Health and Nutrition Examination Survey (NHANES) of the general population 1999–2004 found elevated blood mercury levels in women were associated with higher income and eating more fish [31]. Because high trophic-level fish that have more mercury, such as swordfish, are costly in the U.S, the current project tested the hypothesis that seafood consumption habits are a socioeconomically related risk factor for ALS.

In the U.S. population, fish consumption is the primary source of mercury [5,6,31]. In a San Francisco study of mercury biomarker levels over time (*n* = 67), higher blood mercury levels correlated with swordfish consumption and decreased significantly after they stopped eating fish (*p* < 0.0001) [9]. Nevertheless, our study has corroborated the findings of Parkin Kullman and Pamphlett from an international online questionnaire that similarly did not find a relationship between risk of ALS and either seafood consumption or seafood-based mercury intake [32]. One explanation for these findings is that dietary recall is notoriously difficult, and accurately listing the frequency and species of fish consumed can be challenging. Seafood species choice in the grocery store may be determined by the price-per-pound that week and the appearance or other factors such as a fresh appearance, rather than species, and thus misclassification may be influencing the results of these studies towards the null.

Other sources of mercury exposure are also possible risk factors. Parkin Kullman and Pamphlett also assessed mercury amalgam dental fillings as an alternative source of mercury, but did not find an association with ALS [32]. Mercury is emitted into the air by some industrial activities, yet Palacios et al. found limited evidence of an association between airborne mercury reported to the U.S. EPA National Air Toxics Assessment (NATA) and incidence of the neurodegenerative disease Parkinson’s in a prospective cohort of female nurses (HR 1.33 95% CI 0.98–1.79) [33]. Anti-aging and skin-lightening skin-care products that contain mercury are used and sold in the U.S. illegally, marketed as creams to combat age spots and wrinkles [34].

It is possible that other unmeasured dietary components could be modifying the absorption of mercury via the gastrointestinal tract. In vitro studies demonstrate that absorption of methylmercury through the intestinal epithelium is significantly reduced by cells secreting mucus and by l-cysteine, bile salts, and food components [35]. The intestinal microbiome is also capable of changing the methylation status of mercury, which could also impact the bioavailability and toxicity [36]. A study of twins with discordant socioeconomic deprivation status showed significant differences in the composition of their fecal 16s rRNA microbiomes [37]. Such influences could dissociate our calculated mercury intake via seafood from the absorbed neurotoxic dose, making it a poor estimate of exposure. Similarly, the trace element selenium may also reduce the bioavailability of methylmercury, but because of geographic variation, the selenium-to-mercury molar ratios for the tissue of fish species varies widely [7]. Selenium is also present in other dietary components, making it difficult to estimate its exposure levels using our questionnaire data.

Longitudinal analysis of over one million participants found that greater omega-3 PUFA intake was associated with a reduced risk for ALS [4]. However, our questionnaire-based estimation of omega-3 PUFA consumption via seafood was not associated with ALS. Again, this may be due to the amount of misclassification in self-reported past fish consumption for species and frequency. We also addressed the hypothesis that PUFA would counteract mercury with a negative interaction; however, our data as shown in Figure 4 does not show variation in the mercury–ALS relationship with increasing PUFA quartiles. Using exposure biomarkers, several other studies have found no correlation between omega-3 PUFA and ALS and Alzheimer’s disease neurological impairment. Notably, O’Reilly et al. used gas liquid chromatography to assess PUFA levels in prediagnostic plasma samples archived from large cohorts (with *n* = 275 ALS patients identified). They found no association between total, *n*-3, and *n*-6 PUFA and subsequent ALS risk [38]. Omega-3 PUFA concentration in the blood was also not related to the incidence of dementia and Alzheimer’s disease [39].

The statistically significant factors in our multivariable model of ALS risk were ALS family history and family income. Approximately 5–10% of ALS cases have inherited genetic susceptibilities to the disease, which is similar to the proportion we observed and conferred a ~four-fold increased risk [1]. We observed a positive association between family income and ALS risk. Similarly, a New Jersey study of *n* = 493 ALS cases diagnosed in 2009–2011 found significantly elevated ALS relative risk (RR) of 1.37 (95% CI 1.02–1.82) in the highest income quartile compared to the lowest [40]. The CDC’s National Institute for Occupational Safety and Health (NIOSH) National Occupational Mortality Surveillance (NOMS) study also found that occupations associated with higher socioeconomic status (e.g., computer and mathematical, architecture and engineering, legal, and business operations) had elevated ALS mortality, after accounting for age, sex, and race [41]. Interestingly, with the multivariable model that included adjustment for family income, it was suggested that attending college or graduate school decreased risk, although statistical significance was not reached. The protective effect of longer duration of education was observed in a recent Chinese case–control study (OR = 0.18 95% CI 0.08–0.43) [42]. It is possible that socioeconomically related behavioral risk factors or exposures could modify ALS risk, and future investigation into these factors is warranted.

Limitations of this project include the use of self-reported dietary choices of seafood types and frequencies (e.g., weekly, monthly, and annual). We asked ALS patients to recall their typical fish and seafood consumption 6 months before their ALS diagnosis and asked controls to report on a similar timeframe. These details are difficult to accurately record retrospectively, resulting in some misclassification of our mercury and omega-3 PUFA exposure estimation. Moreover, mercury concentrations for each seafood item are highly variable [14]. Our study included *n* = 165 ALS cases and 2:1 matched controls from the Northern New England and Ohio regions, which may not be generalizable to other populations. We contacted controls from these regions by mail, and the 10% participation rate is a weakness of this approach. Nonetheless, our results are consistent with those of a recent international questionnaire study [32]. The random digit dialing approach obtained a similarly low response rate of 11% when it was used recently to obtain a population-based control group for comparison with the National ALS Registry [43]. Ascertainment bias is unlikely to explain the association between higher income and increased risk ALS, because we observed an inverse relationship with education, with those attending graduate school at reduced ALS risk, compared to those who had a high school education (adjusted *p* = 0.019).

## 5. Conclusions

In summary, our current work does not support fish and seafood consumption as an ALS risk factor using estimates of mercury, and we did not observe a counteracting effect of omega-3 PUFA content of the seafood. This finding leaves open compelling questions regarding the causal factors that mediate the relationship between ALS and income, as well as the source of the elevated nail and hair mercury biomarkers associated with ALS risk.

## Figures and Tables

**Table 1:** Characteristics of ALS cases and controls.

Characteristic	Category	Controls	ALS Cases	Univariate
		*n* = 330 (%)	*n* = 165 (%)	*p*-Value
Sex	female	132 (40.0)	71 (43.0)	0.58
	male	198 (60.0)	94 (57.0)	
Age	<50	23 (7.0)	13 (7.9)	0.96
	50–65	159 (48.2)	76 (46.1)	
	65–75	115 (34.8)	58 (35.2)	
	75+	33 (10.0)	18 (10.9)	
ALS family history	no	323 (97.9)	147 (89.1)	<0.001
	1st or 2nd degree	7 (2.1)	18 (10.9)	
Race	non-white	29 (8.8)	16 (9.7)	0.87
	white	301 (91.2)	149 (90.3)	
Awaji criteria	none	330 (100.0)	0 (0.0)	
	definite		81 (49.1)	
	probable		84 (50.9)	
Smoking	ever	154 (47.1)	90 (56.6)	0.061
	never	173 (52.9)	69 (43.4)	
Education	≤high school	107 (32.8)	62 (40.3)	0.64
	tech	40 (12.3)	17 (11.0)	
	college	99 (30.4)	41 (26.6)	
	postgrad	57 (17.5)	24 (15.6)	
	other	23 (7.1)	10 (6.5)	
Income	<$40,000	132 (29.4)	39 (24.2)	0.24
	$40–60,000	92 (20.5)	26 (16.1)	
	$60–80,000	62 (13.8)	23 (14.3)	
	$80–150,000	109 (24.3)	45 (28.0)	
	$150,000+	54 (12.0)	28 (17.4)	
Consume fish	no	170 (51.5)	79 (49.1)	0.68
	yes	160 (48.5)	82 (50.9)	

**Table 2:** Multivariable model of demographic/lifestyle factors and ALS risk.

Multivariable Model	Category	*p*-Value *	OR *	95% CI	
Smoking	Ever		1.0 (ref)		
	never	0.52	0.88	0.59 -	1.30
ALS family history	No		1.0 (ref)		
	1st or 2nd degree	0.0010	3.93	1.74 -	9.08
Family income	<$40,000	0.078	0.55	0.28 -	1.08
	$40–60,000	0.21	0.64	0.32 -	1.28
	$60–80,000		1.0 (ref)		
	$80–150,000	0.60	1.18	0.64 -	2.22
	$150,000+	0.046	2.09	1.02 -	4.34
Education	≤high school		1.0 (ref)		
	technical school	0.38	0.74	0.37-	1.43
	college	0.12	0.67	0.39 -	1.12
	graduate school	0.06	0.55	0.29 -	1.02
	Other	0.43	0.72	0.30 -	1.61
Race	non-white		1.0 (ref)		
	White	0.90	0.94	0.42 -	2.36

* Adjusted for age, sex, and all factors shown in the table.

**Table 3:** Analysis of relationship between fish-derived mercury, PUFA, and ALS risk.

Among Regular Fish Consumers	Controls	ALS Cases	Univariate
	*n* = 160	*n* = 82	*p*-Value
Estimated fish-derived consumption of:	log mean (SD)	log mean (SD)	
Mercury	2.55 (0.44)	2.53 (0.40)	0.82
PUFA	5.33 (0.45)	5.31 (0.43)	0.74

## Data Availability

Mean mercury and omega-3 PUFA concentrations are provided in Table S1.

## Supplementary Materials

The following are available online at https://www.mdpi.com/article/10.3390/ijerph18094528/s1, Table S1: Mean mercury and omega-3 PUFA concentrations. Table S2: Seafood mercury and omega-3 PUFA mean concentration calculations for broad or vague participant responses.
