# Supplementary material for: Amyotrophic Lateral Sclerosis Risk, Family Income, and Fish Consumption Estimates of Mercury and Omega-3 PUFAs in the United States
Source: Int J Environ Res Public Health. 2021 Apr 24;18(9):4528. doi: 10.3390/ijerph18094528 (PMC8123167; doi:10.3390/ijerph18094528)
Supplement: Supplementary file 1 [file ijerph-18-04528-s001.zip › ijerph-1169463-supplementary.pdf]

International Journal of
Environmental Research and Public Health

MDPI

Supplementary Table 1. Mean mercury and omega-3 PUFA concentrations.

Type	Hg (ppm)	PUFA (ppm)
Ahi	0.27	27.9
Anchovies	0.103	140
Bass	0.288	80
Black_crappie	0.029	11.1
Bluegill	0.09	9.4
Calamari	0.044	40
Canned_tuna	0.179	44
Catfish	0.118	40
Clams	0.028	10
Clams_softshell	0.016	10
Cod	0.087	30
Crab	0.098	40
Crab_cakes	0.098	40
Crappie	0.052	11
Fish	0.083	68
Fish_cakes	0.02	40
Flounder	0.119	20
Grouper	0.417	20
Haddock	0.164	20
Halibut	0.254	70
Herring	0.043	182.5
Lobster	0.153	40
Mackerel	0.586	
Mahi_mahi	0.178	13.9
Mussels	0.028	78.2
Octopus	0.1	18.3
Oysters	0.02	68.8
Perah	0.083	68
Perch	0.133	20
Pollock	0.058	50
Salmon	0.048	190
Sardines	0.079	140
Saugen	0.083	68
Scallops	0.04	36.4
Sea_bass	0.12	80
Shrimp	0.053	50
Smelt	0.08	100
Snapper	0.23	20
Squid	0.044	40
Striped_sea_bass	0.285	80
Steelhead	0.187	60
Sushi	0.249	170.2
Swordfish	0.893	20
Swai	0.018	44
Tilapia	0.019	13.5
Trout	0.187	93.2
Tuna	0.45	150.4
Walleye	0.404	39.7
Whitefish	0.09	180

Supplementary Table 2. Seafood mercury and omega-3 PUFA mean concentration calculations for broad or vague participant responses.

**Participant response**	**Mercury and omega-3 PUFA mean concentration calculation**
“Canned tuna”	Average concentrations for albacore and light canned tuna weighted by their relative frequencies of consumption in the United States
“Crappie”	Average concentrations for black and white crappie weighted by relative frequencies of consumption in the United States
"Fish", "Perah", or "Saugen"	Average concentrations for the ten most consumed seafoods in the United States, weighted by their relative frequencies of consumption in the United States
“Fish cakes”	Concentration for "breaded fish sticks and patties" (mercury) or "fish sticks" (omega-3 PUFA)
“Perch”	Average concentrations for ocean and freshwater perch, weighted by their relative frequencies of consumption in the United States
“Steelhead”	Used mean omega-3 PUFA concentration for trout, which was closest available
“Sushi”	Average concentrations for tuna and salmon, the two most common seafood species found in sushi

Abbreviations: omega-3 PUFA = omega-3 polyunsaturated fatty acids.
